# Announcement: Fourth European Biological Inorganic Chemistry Conference, EUROBIC 4

**DOI:** 10.1155/MBD.1998.119

**Published:** 1998


					FOURTH
INORGANIC
EUROBIC
14o
EUROPEAN B LOGICAL
CHEMISTRY CONFERENCE
July 20-25, 1998
Seville, Spain
Organizing Committee
Chairman: F. Gonzalez-Vilchez (Sevilla)
Secretary: R. Vilaplana (Sevilla)
Treasurer: A.J. Palomares (Sevilla)
Members: M. Acosta-Echevarria (Murcia), C. Alonso (Madrid), A. Bernalte (Badajoz), J. Borras
(Valencia), G. Garcia-Herdugo (Sevilla), P. Gonzalez-Duarte (Barcelona), C. Gomez-Moreno
(Zaragoza), M.A. de la Rosa (Sevilla), J.M. Salas (Granada), J. Sordo (Santiago), A. Terron (Palma de
Mallorca)
International Steering Committee
F. Gonzalez-Vilchez (Spain, Chairperson), B. Meunier (France), J. Reedijk (The Netherlands), M.A.
de la Rosa (Spain), C. Veeger (The Netherlands), R. Weiss (France), R. Thorneley (UK, Secretary)
Introduction
EUROBIC 4 is the fourth edition of the successful european bioinorganic chemistry conferences
previously celebrated in Newcastle (UK), Florence (Italy) and Noordwijkerhout (The Netherlands).
The conference is an excellent and wide international forum for the interchange of scientific
contributions and ideas on all aspects of Biological Inorganic Chemistry and for the advancement of
high quality science in this internationally recognized area.
The conference is organized under the auspices of the European Science Foundation (ESF)
Programme "Chemistry of Metals in Biological Systems" (METBIO), The Ministery of Education and
Culture (Spain), The Education Councillorship of "Junta de Andalucia", The Royal Spanish
Chemical Society and the University of Sevilla (Spain).
Location and dates of celebration
EUROBIC 4 will be held in Sevilla (Spain), on 20th (arriving day) to 26th (departure day) of July,
1998. Sevilla, the capital of Andalusia, is one of the most beautiful and historical cities of Western
Europe, founded over three thousand years ago and capital of old civilizations as Tartessians,
Carthaginians, Romans and Arabs. Monuments as the Gothic Cathedral with the famous arab tower
119
"La Giralda", the Alcazar, the Archive of Indias and the Gold Tower are some of the many
architecture jewels that combines their splendid beauty with modern buildings and infrastructures
constituting the new city. It boasts a large, prestigious university, several high-technology research
centers belonging to the Spanish Council of Scientific Research and many cultural attractions.
Sevilla, linked to Madrid by an economic high-speed train, is also easily accessible by air from many
european airports, rail and roads.
The Organizing Committee is very pleased to invite all members of the bioinorganic community to
attend and participate in EUROBIC 4.
The magnificent Hotel Alcora has been chosen as the appropriate sede for the Conference.
Located at the high part (Aljarafe) of the very next surroundings of Sevilla, this luxury hotel posses
a recognized tradition as Congress Centre.
Accompanying persons would be the best welcome. The sunny and pleasant climate of July, the
proximity of historical cities such as Granada and Cordoba and the opportunity to visit the recent
and already famous attraction "lsla Magica" located in the former site of the 1992 Universal
Exposition guarantees a happy and nice stay during the Eurobic 4.
Congress Programme
The programme includes plenary lectures, invited oral contributions in sessions and minisymposia
and two separate poster sessions. The posters will constitute the important and integral part of the
conference and scientific contributions in this form are strongly solicited from all participa nts.
The organization encourages the participation of young scientists and has stablished a reduced
registration fee for graduate students and postdoctoral researchers presenting contributions. All
participants will be actively involved in the programme either as lecturers, discussion leaders or as
presenters of posters.
The third European Medal for Bioinorganic Chemistry to an outstanding young scientist in this
branch of Science will be awarded and the laureate invited to give a plenary lecture.
Scope of the Conference
EUROBIC 4 will focus on the following themes on Biological Inorganic Chemistry:
Metalloproteins (Two sessions).
Metals in Medicine (Two Sessions).
Electron and proton transfer.
Metals and the Environment.
Metals and Agriculture.
Theoretical studies and models.
Minisymposia will be dedicated to the following topics:
giosensors.
MetaI-DNA interactions.
Vanadium proteins.
Biological role of peroxidases.
Biomineralization.
Metallothioneins.
Language
English is the official language of the Conference.
Following scientists have accepted invitation to participate:
Plenary Lecturers
H. Sigel (Switzerland): Opening Lecture
L. Banci (Italy)
L. Casella (Italy)
L.J. Krishtalik (Russia)
B. Lippert (Germany)
P. Siegbahn (Sweden)
C. Veeger (The Netherlands)
G. Canters (The Netherlands)
M.J. Clarke (USA)
M. Leng (France)
V. de Lorenzo (Spain)
J. Ulstrup (Denmark)
A. Xavier (Portugal)
F. Amstrong (UK): Winner of the 3rd European Medal of Bioinorganic Chemistry
120
Some Invited Participants
K.K. Andersson (Norway)
V. Brabec (Czech Republik)
V. Conte (Italy)
M.C. Feiters (The Netherlands)
C.D. Garner (UK)
C. Gotor (Spain)
W.R. Hagen (The Netherlands)
H. Kozlowski (Poland)
C. Luchinat (Italy)
G. Mendoza (Mexico)
V. Moreno (Spain)
G. Natile (Italy)
B. Norden (Sweden)
J.N. Rodriguez (Spain)
J. Salgado (Spain)
B. Spiess (France)
A.G. Sykes (UK)
T. Theophanides (Greece)
D. Turner (UK)
M. Vasak (Switzerland)
R. Weiss (France)
I. Bertini (Italy)
R. Cammack (UK)
R.R. Crichton (Belgium)
S. Forsen (Sweden)
C. Geraldes (Portugal)
N. Hadjiliadis (Greece)
J. Kozelka (France)
P. Kroneck (Germany)
M. Medina (Spain)
L. Messori (Italy)
G. Moore (UK)
W. Nitschke (France)
J. Reedijk (The Netherlands)
M.J. Romao (Portugal)
M.A. Santos (Portugal)
I. Sovago (Hungary)
M. Teixeira (Portugal)
R. Thorneley (UK)
R. Van Eldik (Germany)
A. Vila (Argentina)
R.J.P. Williams (UK)
Registration
Participants
A) Registration fee
before April 30, 1998: Members of SBIC: 35,000 ptas. Non members: 41,000 ptas.
afterApril30, 1998: Members of SBIC: 44,000 ptas. Non members: 50,000 ptas.
Registration fee covers scientific participation and conference documents, coffee breaks and
welcome reception.
B) Lunches (special package of five lunches): 13,400 ptas.
C) Social programme: includes guided visit to the city, excursion and closing banquet: 15,000 ptas.
Accomodation
D) Special package of six nights:
35,000 ptas. (per person in a twin room with double occupancy)
55,000 ptas. (per person in a single occupied room).
Concepts B, C and D are optionals for participants and accompanying persons.
A reduced conference fee (20,000 ptas.) would be awarded by the Organizing Committee to a
limited number of young participants (under 35) presenting a poster. An application letter, a brief
CV (one page) and a recommendation letter from a senior scientist should be addressed to Dr. R.
Vilaplana before 31st of May, 1998. Those awarded young researchers would be refunded at the
Congress site. The organizers are trying to get funds to enable additional reduced accomodation
fees for young researchers.
2) Accompanying, persons
Registration: 25,000 ptas.
It includes documents, welcome reception and an additional guided visit to the moorish palace "El
Alcazar" and the gothic Cathedral of Seville. Lunch is included in the visit. Tickets for lunches might
be adquired during the Congress. Optional visits may be booked at Congress site.
121
Payment
Payment should be made as follows:
A) Registration (concepts A, B and C).
1) By bank transfer to: Account no.: 0030 8443 95 0850377273
Congreso Eurobic-4
BANESTO, Urb. Reina Mercedes
Avd. Reina Mercedes, 27
41012 Sevilla (Spain)
2) By draft check payable to "EUROBIC 4"
Draft check or copy of the bank transfer should be sent together with the REGISTRATION FORM
to:
Dr. R. Vilaplana
Departamento de Ctuimica Inorganica
Facultad de Ctuimica
Aptdo. 553
41071 SEVILLA (Spain)
who will send additional information like REGISTRATION FORM and ACCOMODATION FORM to
those who are interested.
Make sure that your name and address are clearly stated on all payments and transfer documents.
B) Accomodation (concept D)
1) By draft check payable to "ECUADOR CONGRESS"
2) By Credit cards:
All major credit cards such as Eurocard, Master Card, American Express or Visa may be used for
payment. Please, fill in your card number and expiration date on the ACCOMODATION FORM.
All remittances received will be acknowledged.
Cancellation
Cancellations received before May 31, 1998 will be accepted with a partial refund of 70%. It is not
possible to offer any refunds after that date.
122

				

